# Rotating magnetic field as tool for enhancing enzymes properties - laccase case study

**DOI:** 10.1038/s41598-019-39198-y

**Published:** 2019-03-06

**Authors:** Agata Wasak, Radosław Drozd, Dorota Jankowiak, Rafa Rakoczy

**Affiliations:** 1 West Pomeranian University of Technology Szczecin, Department of Immunology, Microbiology and Physiological Chemistry, Piastów Avenue 45, 70–311 Szczecin, Poland; 2 West Pomeranian University of Technology Szczecin, Faculty of Chemical Technology and Engineering, Institute of Chemical Engineering and Environmental Protection Processes, Piastów Avenue 42, 71–065 Szczecin, Poland

## Abstract

The aim of this study was to analyse the effect of rotating magnetic field (RMF) exposition on the fungal laccase catalytic properties. The results obtained in the study revealed that RMF may positively alter the laccase activity. A significant increase in activities of 11%, 11%, and 9% were observed at 10 Hz, 40 Hz and 50 Hz, respectively. Exposure of laccase to the rotating magnetic field resulted in its increased activity at broader pH range and a slight shift in optimum pH from 4.0 to 4.5 at RMF with frequency 20 Hz. The results show that the enzyme activity, stability, and optimum pH can be significantly altered depending on the characteristic of the applied RMF. Application of rotating magnetic field opens a new way for controlling and directions of enzyme-based bioprocessing.

## Introduction

Numerous industry branches use enzymatic processes because, compared to chemical catalysts, they are rapid, carry out specific chemical transformations, save reagents as well as energy^1,2^. The nature of designed enzymes to catalyse chemical reactions to be highly selective and efficient in terms of energy saving^3^. Despite the fact that enzymes are enormously applicable, their use in industrial processes on a large scale is hampered in many cases by their poor operational stability and low resistance to the process conditions. At present, the main research is focused on protein engineering solutions; unfortunately their use is limited due to insufficient knowledge about the enzyme structure and its mechanism of action^4,5^. The common technique used widely for enhancement of enzyme activity and operational stability is immobilization them on various supports. Immobilized enzymes thanks to interaction with carrier structure are usually more resistant for unfavourable reaction conditions like to low or to high temperature, pH, ionic strange and also occurrence of ligands non-organic and organic origin that could destabilize molecular structure of biocatalyst and affect its performance^6,7^. Using physical factors such as electromagnetic field (EMF) and magnetic field (MF) to stimulate and alter enzyme activity and its catalytic properties, may turn out to be an attractive alternative^8^. A potential MF effect on reactions catalysed by enzyme opens up new opportunities to alter their direction as well as kinetics^9^. The external MF applied may affect the molecular structure of a given enzyme, thus modifying its catalytic properties^10^. Moreover, MF exposure may also affect the kinetic energy of unpaired electrons released in the course of catalytic act, which may have an impact on chemical reactions and biological processes. Generally, MFs can be divided into direct-current magnetic field (DCMF) and alternating current magnetic field (ACMF). The DCMF does not vary in time or it changes very slowly. The ACMF oscillates in the direction of frequency. It should be noticed that typical example of this kind of MF is a rotating magnetic field (RMF). This type of MF, which arises as a resultant field during the superposition of two or more ACMFs of identical frequency but spatially displaced in phase with respect to one another. It should be noticed that the RMF may be considered an electromagnetic stirrer. The application of this kind of magnetic field involves the induction of eddy currents in aqueous media, which creates their own magnetic field being in a co-action with the principle one (that is used for the exposure). This cooperation of magnetic fields can enhance the mass transfer by reducing the diffusion mass transfer resistance^11^.

To date, the studies have been mainly focused on the effects of static magnetic field on the activity of: α-amylase, superoxide dismutase, horseradish peroxidase or tyrosinase as well as the effects of rotational magnetic field on the lipase and chitinase activity were tested. However, an unequal impact of MF on analysed enzymes was observed, that can be connected with different reaction course of catalysed reaction and obvious structural difference of examined enzymes. Moreover, the inactivation of native peroxidase as opposed to the immobilised counterpart that had been immobilized by entrapment in gelatine membranes or covalent attachment to nylon membranes, was reported under the same research conditions (50 Hz, 1 mT, extremely low frequency (ELF) of magnetic field). The authors speculated that the immobilised peroxidase does not reflect the actual enzyme-EMF interaction and *in vivo* catalyst activity. The discrepancies in observed activity of the biocatalysts exposed to EMF were caused by: the type of applied magnetic field and its characteristics (frequency, magnetic induction), time of exposure, enzyme type, and its form (native, immobilized) as well as the test system (*in vivo or* *in vitro)*^12^–^14^. Unfortunately, the mechanisms, due to which MF affects the enzyme activity and its catalytic properties, have not been recognised yet.

Laccases (EC 1.10.3.2; *p*-diphenol; benzenediol oxygen oxidoreductase) are multi-copper enzymes that catalyse the oxidation of phenolic and non-phenolic compounds, including lignin and environmental pollutants, using molecular oxygen as a terminal electron acceptor and producing water as the only by-product^15^. Due to such attractive properties, laccase was successfully used in: bio-pulping and bio-bleaching in the textile industry, food industry as a biosensor component, delignification, bioremediation, biodegradation, organic synthesis, biofuels production^16,17^. Despite great potential of laccase, its use on a large scale is hampered by its low activity, operational stability and sensitivity to the environmental factors such as temperature and pH^18^. Therefore, any alternative methods or factors contributing to enhanced activity, operational stability and tolerance to temperature and pH of laccase are being constantly searched for^19^. For instance, the group of Xia *et al*.^20^ used the alternating magnetic field to intensify the mixing and oxidation rate of catechol catalysed by laccase immobilized on magnetic nanoparticles. Unfortunately, there are no data in the available literature on the effect of magnetic field on native laccase. Moreover, most studies upon enzymes exposed to MF, both *in vivo* and *in vitro*, focus on their activity only, while not testing their basic properties and catalytic parameters such as: K_M_ and V_max_ or stability.

In this paper, for first time we report studies on the effect of rotating magnetic field (RMF) on activity, kinetic parameters and pH stability of laccase from *Trametes versicolor*. The effect on laccase exposed to different low frequencies (10–50 Hz), magnetic inductions (15–18.5 mT) for various periods of time, was examined.

## Results and Discussion

The effect of low-frequency RMF (10–50 Hz) on a native laccase activity and its basic catalytic parameters were analysed. Depending on RMF parameters, the rotating magnetic field has different characteristics that could result in unlike catalytic behaviour of analysed enzyme. The available literature reports mainly about the analysis of living cells enzymatic systems response on the exposure to various types of MF. Considering the influence of various kinds of magnetic field on activity and structural properties of purified enzymes, only a few reports were published and there are scarce reports on using the static field^8^,^21^.

### Effect of RMF on laccase activity

**Figure 1:**
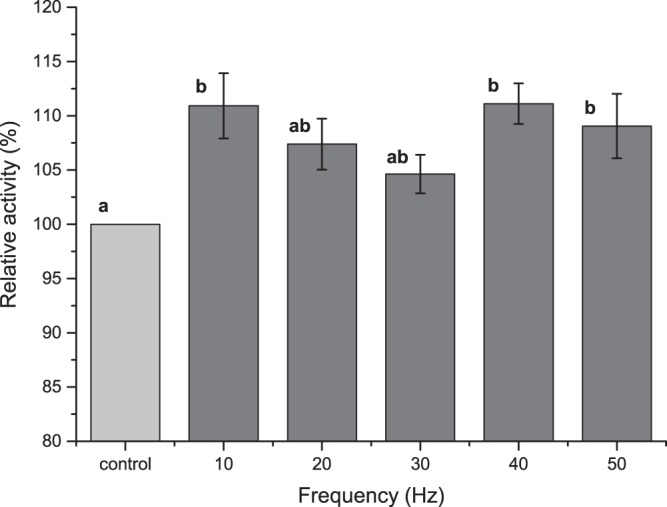
Effect of different RMF frequencies on the laccase activity (reaction time 2 minutes, 0.5 mmol ABTS, pH 4.0. a, b - means sharing the same superscript are not significantly different from each other (*p* > 0.05).

We investigated the effect of rotating magnetic field on laccase activity for a range of frequencies (10–50 Hz) and magnetic inductions (15–18.5 mT) (Fig. 1**)**. A varied effect of different frequency RMF on the laccase activity was observed. A significant increase (*p* < 0.05) in laccase activity (11%, 11% and 9%) was observed for samples exposed to frequencies of 10, 40 and 50 Hz, respectively. At the frequency of 20 Hz, the increase in activity was similar to the significance level (7%), while exposing samples to a frequency of 30 Hz did not produce any change in that parameter. Based on the obtained results, we can state that any changes in activity were frequency dependent, which in turn had an effect on magnetic induction. It may result from the fact that exposure to various RMF frequencies causes various resonance levels of enzyme structure. It is common knowledge that electromagnetic fields affect the proteins dynamically as well as the metal ions in their structure^8^. Rotating magnetic fields provided by public suppliers (50 Hz) probably have no negative effect on the laccase structure as no decrease in the enzyme activity was observed in all the samples (Fig. 1). However, it was observed that low frequency electromagnetic field (ELF-EMF) (50 Hz, 1.8 mT) as well as static magnetic field (SMF) (200 mT) may dampen the stretching vibration in the C=O and C-N bonds as well as NH bent bonds may change the protein secondary structure^9^. The respective results were obtained by Xia *et al*.^20^ and Mizuki *et al*.^13^ but they did not observe any changes in the laccase activity under the alternating magnetic field of 600 Hz (1.0 mT) and lipase, chitinase activity in the rotational magnetic field (9.55 kA m^−1^). These discrepancies may result from applying different frequencies and the type/characteristics of the field.

### RMF effect on laccase kinetic constans V_max_ and K_M_

There were no significant changes in K_M_ with one exception when laccase was exposed to the frequency of 30 Hz, then K_M_ increased from 0.021 mmol to 0.056 mmol (Table 1).

**Table 1:** Kinetic constant of laccase exposed to RMF with ABTS as a substrate at pH 4.0. ^a^Means sharing the same superscript are not significantly different from each other, ± - means standard deviation.

RMF frequencies	K_M_ [mmol]	V_max_ [µmol × L^−1^ min^−1^]	V_max_/K_M_
Control	0.021 ± 0.001	1.30 ± 0.01	49.60 ± 4.0
10 Hz	0.020 ± 0.001^a^	2.57 ± 0.11^a^	142.4 ± 6.5^a^
20 Hz	0.010 ± 0.002^a^	2.19 ± 0.04^a^	169.4 ± 21.7
30 Hz	0.056 ± 0.014	3.18 ± 0.11^a^	57.20 ± 9.1^a^
40 Hz	0.020 ± 0.001^a^	3.45 ± 0.04^a^	220.70 ± 19.0^a^
50 Hz	0.020 ± 0.001^a^	3.46 ± 0.14	147.3 ± 3.5^a^

Catalytic constant of laccase exposed to RMF with ABTS as a substrate at pH 4.0. ^a^Means sharing the same superscript are not significantly different from each other (*p* > 0.05), ± - means standard deviation.

**Figure 2:**
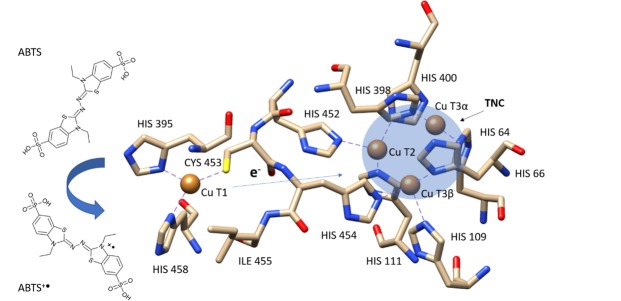
Schematic representation of ABTS oxidation catalysed by laccase. The figure was prepared according to *Trametes versicolor* laccase 3D structure (PDB code 1 kya).

While, comparing to the control, the increase in laccase maximal reaction velocity was observed at all tested frequencies; the highest one was at 50 Hz (3.46 µmol × L^−1^ min^−1^) and the lowest at 20 Hz (2.19 µmol × L^−1^ min^−1^). The highest efficiency of enzyme catalysis (V_max_/K_M_) was observed at 40 Hz, the lowest one at 30 Hz and it was similar to the control. The RMF characteristic changes as time function is specific and its characteristic depends on the frequency of alternating current. However no simple linear relationship was found between the increased an alternating current (AC) frequency and changes in K_M_ and V_max_. For RMF induced by AC with low frequencies, fluctuations in magnetic induction (B) value were grater compare to higher frequencies. However the average B value for RMF induced in all analysed AC frequencies not differ significantly, but with tendency to constant growing up. This can partially explain lack of significant difference in maximal velocity at RMF with frequencies from 10 Hz to 40 Hz. The strength and periodical changes of RMF properties can probably be a factor that can determine the changes of maximal velocity and substrate affinity constants that was manifested by variation in K_M_/V_max_ ratio for laccase. Different trend were observed when α-amylase was exposed to SMF, then decrease in K_M_ and V_max_ with a simultaneous increase in the SMF intensity was observed. The changes in K_M_ reflecting the enzyme affinity to a substrate suggest that, depending on frequency, RMF may result in the increased or decreased efficiency of the bond between substrate and enzyme. Different results obtained for various enzymes may result from their specific structural properties and different catalytic activity^8^. Magnetic field can also change the physicochemical properties (e.g. conductivity, dielectric constant) a reaction medium and alter enzyme catalytic properties. A number of enzymes, including laccase, contain metal ions such as: Mg^2+^, Cu^2+^, Fe^2+^, Mn^2+^ and Ca^2+^ incorporated in their structure. They may play a crucial role in stabilising the 3D enzyme structure as well as in the catalytic action^22,23^. Ions are incorporated into the protein structure, they produce ionic, covalent and hydrogen bonds that determine the pathways of electron transfer to target them at the catalytic site of the enzyme^24^. The complex spatial structure of the protein allows for maintaining the specific and unique geometry of the metal sites^25^. The interaction of external magnetic fields with the 3D structure of a protein can potentially induce changes, which results in different geometry of the metal sites in enzyme active site. In metaloproteins, there are semi-polar bonds between metal atoms and histidine in the case of laccase (Fig. 2). Many of them are transition metals, therefore they may be paramagnetic or diamagnetic, thus they can be attracted by external magnetic field. There are four Cu^2+^ atoms present in the laccase active centre, that are located in the binding pocket in a very conservative manner for many multi-copper oxidases (MCOs). First of them, Type 1 (T1) is responsible for oxidising a substrate as well as transferring the intra- and intermolecular electrons along with cysteine, which is present nearby, to three atoms located in the tri-nuclear copper cluster (TNC); Cu Type 2 (T2) and double nuclear Type 3 (T3). TNC reduces O_2_ to water^26^–^29^. Taking into consideration the mechanism of oxidation catalysis (ABTS) by laccase (Fig. 2), the RMF effect may be solved on two levels. The first stage of substrate oxidation depends on CuT1 ion, come from electrons are transferred to T2 and T3 responsible for binding O_2_ and its further reduction to water, which in turn, is essential to T1 regeneration and initiating another catalytic cycle. The geometry of enzyme catalytic centre resulting from the protein structure affects directly the electron and proton transfer both to and from T1 and TNC due to the location of specific amino acid groups^28^. Due to different location and binding amino acids from the catalytic site, the Cu^2+^ ions in the active laccase centre may potentially react in different ways to the external magnetic field and alter their properties^29^. Moreover, the geometric and electron state of other enzymes in their vicinity may affect the properties of certain places in enzyme through spin-spin interactions^30^. In the classical axial ligand, the CuT1-S (thioethen) bond in the blue copper enzyme is in exactly the same place where a substrate was reduced. The change in the properties of blue copper sites, e.g. by the effect of external factors, including magnetic field, may cause changes in its geometry as well as in the electric state of ligand. Unlike other blue copper enzymes such as plastocyanin, fungi laccase belong to the so-called nonaxially liganded T1 sites. The lack of any axial interaction in the T1 laccase atom results in shifting the bond plane toward N(His)-S(Cys)-N(His) and it is compensated by increased covalent bond in CuT1-S (thioethen)^24^. Regardless of the transition state, the T3 atoms in TNC are diamagnetic^31^, which means that the external magnetic field may “repel” them due to their arrangement of electrons. While, the T1 and T2 atoms are paramagnetic, which means that the external magnetic field “attracts” them. The diamagnetic materials, e.g. proteins, may alter their shape and exhibit anisotropic properties in the strong magnetic field^32^. However, the research carried out on diamagnetic materials to date show their poor susceptibility to the external magnetic field - its magnetic induction is not high enough. Most likely, changes in laccase activity exposed to RMF are caused by direct effect of magnetic field on Cu^2+^ ions or altering the protein configuration, which may be indirectly related to the operation of active enzyme site. The studies on the oxidized and reduced states of blue copper sites are based on spotting differences in their spectral properties. However, their crystal forms and model complexes are mostly studied, instead of entire proteins, therefore the site operation is not known in details^24^. Kimura *et al*.^33^ showed that comparing to SMF, even RMF with low magnetic induction may have the same effect on biomolecules. The laccase exposure to RMF may potentially affect the structure of its catalytic site, where the TNC cluster coordinated by eight histidine radicals is suspended^28^. Its protein medium may significantly alter the reduction potential (E_0_) of metal sites by a specific effect of loaded and polar radicals and hydrogen bonds that may stabilise selectively reduced or oxidized sites^24^. When electrons are transferred from an oxidised substrate to the TNC cluster, and further to reduced oxygen molecule, the geometry of T2 location towards T3 plays a key role^34^. Exposing the diamagnetic component of TNC cluster to RMF may also alter (accelerate) the magnetic spin moment of atoms it includes as well as affect the reaction rate of oxygen reduction^26^,^33^. For analysed laccase, it could be probably reason of increase an enzyme maximal velocity at RMF with specific frequencies. Moreover, altered enzymatic activity may be caused by the electromagnetic field affecting the transferred charges, e.g. in the catalytic act, which may affect the changes in the rate of catalysed reaction^11^. When Büyükuslu *et al*.^21^ and Çelik *et al*.^35^ analysed the SMF effect on superoxide dismutase, they suggested that the increased enzyme activity caused by the external magnetic field may be related with increased energy of unpaired electrons and transferring them to the next components of the reaction chain, thus changing the rate of the reaction itself. Magnetic field makes electrons to rotate, due to which they change their singlet state (S) conformation to the triplet state (T) one, and thus free radicals are produced. Thanks to the above process, enzymes exposed to MF may indirectly affect the chemical reactions and biological processes^14^.

### Effect of RMF on optimum and pH stability of laccase

**Figure 3:**
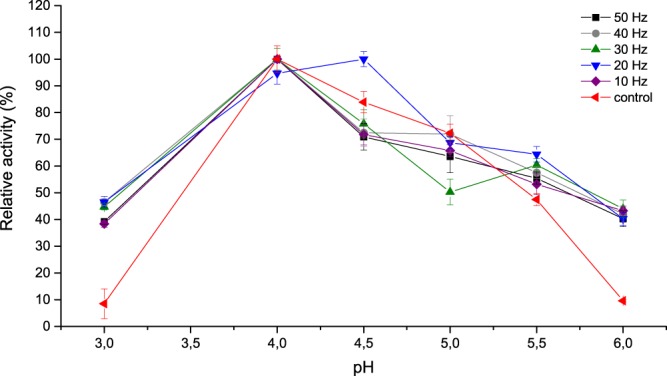
RMF effect on laccase catalytic optimum pH (reaction time 2 minutes, 0.5 mmol ABTS, Tris-glicyne, acetate, and phosphate buffers, 50 mmol).

**Figure 4:**
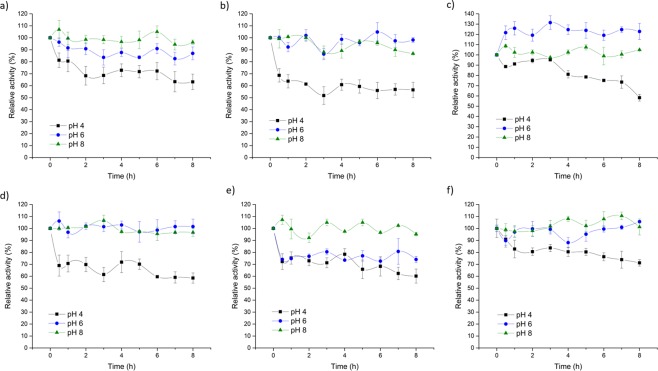
Effects RMF on laccase stability at pH 4.0, 6.0, 8.0, during 8 h incubation under a frequency; (**a**) 10 Hz, (**b**) 20 Hz, (**c**) 30 Hz, (**d**) 40 Hz, (**e**) 50 Hz and (**f**) the control. The initial activity of laccase has been taken as 100%.

The laccase activity under RMF was measured with different pH value in the range from 3.0 to 6.0 at 30 °C. As shown in Fig. 3, both control (not exposed) and exposed enzyme exhibited similar profile in the pH range from 4.0 to 5.5 with the highest activity at pH 4.0. A shift to pH 4.5 was observed only at 20 Hz. Exposure to RMF did not significantly affect the optimum pH for enzyme, however increased laccase activity was observed at all pH values under study, particularly at pH 3.0 and pH 6.0. When laccase was exposed to all frequencies, its activity increased by 30–36% was observed at pH 3.0 and by 30–34% at pH 6.0. The phenomena occurring in water and water solutions exposed to MF and EMF are called “magnetic memory effect”. The changes depend on the following parameters: electric field intensity, direction of magnetic field lines, time of exposure, pH, water flow rate or the type of dissolved substances^35–37^. The pH analysis carried out under RMF exposure did not show, however, any significant changes in pH of buffers used under exposure (unpublished data); hence that factor may be excluded as a potential cause of observed differences in the laccase activity. Exposure to SMF may induce changes in the network of hydrogen bonds and water polarisation^37^,^38^. According to Pang and Deng^37^, SMF only affects the changes in distribution and polarisation of molecules, while not in the constitution of water. Lack of differences in pH of applied buffers may be related to different nature of applied field and low RMF magnetic induction (15–18.5 mT). According to Szcześ *et al*.^39^, taking the quantum field theory into account, SMF magnetic induction of 15 mT is not enough to get in resonance with a rotational state of water. The time of exposure of buffers to RMF was not the factor affecting the changes in pH. No changes in the pH level were observed after 2 min, 1 h and 8 h of RMF incubation (unpublished data). The effect of different RMF frequencies and pH on the laccase activity was tested **(**Fig. 4**)**. The enzyme in relevant buffer (pH 4.0 acetate buffer, 6.0 and 8.0 phosphate buffers, 50 mmol) was incubated in RMF at different frequencies for 8 h, a sample was taken every 1 h and laccase activity was measured. The control samples were not exposed to RMF (Fig. 4f). When laccase was incubated at pH 4.0 and exposed to all above frequencies, a slight decrease (12%) in its activity compared to the control was observed. However, significant decrease (20%) in the laccase activity was observed at 50 Hz after the first hour of incubation (Fig. 4e). For samples incubated at 10 Hz, minor fluctuations in the laccase activity were observed in further measurements or the most homogeneous stability profiles were observed at all pH values **(**Fig. 4a). It may suggest a slight effect of certain RMF frequencies on the laccase structure, thus its stability. Compared to the control, in samples incubated at pH 6.0 at 50 Hz (Fig. 4e) and 30 Hz (Fig. 4c), significant decrease and increase in activity (31% and 17%, respectively) after 8 h was observed. Comparing to the control, at 50 Hz and 10 Hz, laccase was the most stable at pH 8.0, for 30 Hz at pH 6.0, while laccase stability at pH 6.0 and 8.0 exposed to 20 Hz and 40 Hz was similar.

## Conclusions

The concept based on applying the bioreactors supported by RMF to control an enzymatic reaction seems to be a promising method to modify the bioprocesses based on enzymes. The effective enhancing catalytic power of enzyme by applying physical or chemical factors requires maintaining their high operational stability. We showed that the laccase activity can be modified by the exposure to external magnetic field without negative impact on the enzyme stability. However, in order to explain the reasons behind changes in the laccase activity regardless of the observed RMF effect, further studies are needed.

## Material and Methods

### Chemicals

ABTS (2,2′-azino-bis (3-ethylbenzthiazoline-6-sulfonate) was purchased from Sigma-Aldrich Chem. Co. (St. Louis, MO, USA). All other chemicals were of analytical grade.

### Fungal strain and culture maintenance

The strain of *Trametes versicolor* was from the culture collection of the Department of Immunology, Microbiology and Physiological Chemistry, West Pomeranian University of Technology, Szczecin (Poland); it was deposited with ID number 5/22/09/14 there. The fungus was isolated from decayed wood in the region of Szczecin and maintained through periodic transfer at 4 °C on potato dextrose agar plates (PDA, Difco). Subsequently, the fungus mycelium was transferred from slants to plates and incubated at 25 °C for 14 days, then it was used for inoculation.

### Laccase purification

The liquid medium (5 g/L potato extract, 10 g/L glucose, 0.1 g/L MgSO_4_, 0.6 g/L KH_2_PO_4_; pH 6.0) was inoculated with agar plugs. Cultures of 5/22/09/14 were grown in 1 L Erlenmeyer flasks under stationary conditions at 25 °C for 10 days. After a 7-day cultivation, CuSO_4_ (final concentration of 1 mmol) was added in order to enhance laccase production. Then, the broth was filtered (Whatman No. 1 paper), and the supernatant thus obtained was lyophilized and stored at −20 °C until the extraction procedure. The lyophilizate was dissolved in phosphate buffer (10 mM, pH 7.0) and further purified by three steps of three phase partitioning (TTP) with ammonium sulphate precipitation (20–70%) and *tetr*-butanol, ion exchange chromatography on HiTrap DEAE-Sepharose (10 mmol phosphate buffer, pH 7.0) and HiTrap ANX Sepharose (10 mmol phosphate buffer, pH 7.0) eluting a linear gradient (0–0.25 mmol NaCl in 200 mL buffer) at 1 mL/min. The fractions with significant activity of laccase were pooled, dialyzed, and applied to gel filtration chromatography by fast protein liquid chromatography (FPLC) on Sephacryl HR-300 (10 mmol phosphate buffer with added 10 mmol NaCl, pH 7.0). All purification steps using liquid chromatography were carried out by means of ÄKTA Purifer system (Amersham Bioscience).

Protein determinations were performed according to the method by Bradford^40^ using bovine albumin as the standard.

### Experimental set-up (RMF generator)

**Figure 5:**
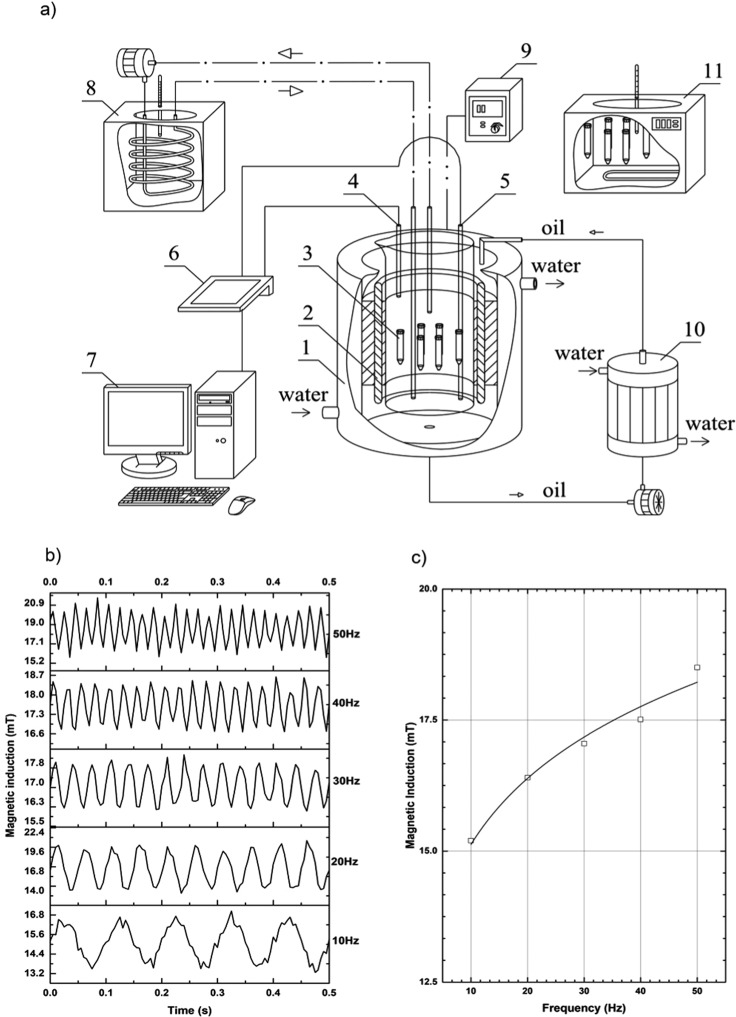
(**a**) Schematic diagram of experimental set-up: 1 – cooling jacket, 2 – RMF generator, 3 – test tube, 4 and 5 – microprocessor temperature sensors, 6 – multifunctional computer meter, 7 – personal computer, 8 – thermostat with a circulating pump, 9 – AC transistorised inverter, 10 – heat exchanger, 11 – thermostat with a control tube; (**b**) changes in recorded magnetic induction over time for selected RMF frequencies; (**c**) changes in mean magnetic induction depending on RMF frequency.

A schematic diagram of the experimental device employed in the present study is shown in Fig. 5a. The experimental set-up included the following components: cooling jacket (1), RMF generator (2), glass container with a test tube (3), AC transistorised inverter (9), computer (7) equipped with the software to control the RMF generator, microprocessor sensor (4 and 5). The RMF generator consisted of a three-phase stator with an induction squirrel cage motor. The windings powered by alternating current generated the rotating magnetic field (RMF) with constant angular frequency. In our experiment, the stator was supplied with 50 Hz three-phase alternating current. The frequency was changed by an AC transistorised inverter (9) (Commander SK, Nidec Industrial Automation UK Ltd). A glass container filled with demineralised water was used for water bath incubation of test tubes (3). It was axially aligned with the RMF generator and its lower and upper ends were positioned symmetrically. The incubation temperature during the laccase exposure to RMF was controlled by a thermostat (8) (UTU-3, ZEAMiL, Poland) with a circulating pump (WZ-250/BY, Ożarów Mazowiecki, Poland) and a cooling jacket (1). The system maintained constant water flow and set constant temperature of water bath. The temperature fluctuations in the flask were measured by microprocessor temperature sensors (4 and 5) (LM-61B, National Semiconductor Corporation, USA) connected to a multifunction computer meter (6) in order to ensure constant temperature of 30 °C.

### Rotating magnetic field - characteristics

RMF with the magnetic induction (*B)*, was controlled by the alternating current frequency. In the experiment procedure, the frequency varied from 10 to 50 Hz. Microprocessor magnetic sensors connected to the Hall probe (Smart Magnetic Sensor-102, Asonik, Poznań, Poland) and a computer detected magnetic induction in a flask at different measurement points. The electrode was calibrated in zero Tesla chamber before taking every measurement. Next, it was transferred to an Eppendorf tube filled with acetate buffer at pH 4.0 (50 mmol) and an teslameter was switched on. Induction measurement was recorded for 2 min in the buffer with optimum pH for laccase activity and then repeated four times. Subsequently, the obtained results were averaged and presented in Fig. 5b.

Based on the obtained results, mean magnetic induction depending on RMF frequency was determined in order to describe the applied magnetic field. Therefore, the changes in time at different frequencies were determined according to the following analytical relation (equation 1):

1 $$B(t)={p}_{1}+{p}_{2}(\pi \frac{t-{p}_{3}}{{p}_{4}})$$

where:

*B* - magnetic induction, mT;

*p*_1_, *p*_2_, *p*_3_, *p*_4_ - parameters of analytical relationship;

*t* - time, s.

Next, mean values for each measurement were determined by the following equation 2:

2 $$\langle B\rangle ={\int }_{0}^{t}[{p}_{1}+{p}_{2}\,\sin (\pi \frac{t-{p}_{3}}{{p}_{4}})]dt\Rightarrow \langle B\rangle =\frac{1}{\pi }[{p}_{2}{p}_{4}\,\cos (\frac{\pi {p}_{3}-\pi t}{{p}_{4}})-\pi {p}_{1}t]$$

Computed mean values of magnetic induction are shown in Fig. 5c.

### Laccase activity assay

The reaction was carried out in the standard assay conditions, namely: 50 mmol acetate buffer (pH 4.0), 0.5 mmol ABTS as a substrate and increase absorbance at 420 nm (ε = 36.000 mol^−1^ cm^−1^) was monitored for 2 min at 30 °C. ABTS oxidation was measured spectrophotometrically by an Infinite 200 PRO NanoQuant microplate reader (Tecan, Männedorf, Switzerland). One unit of enzyme activity (U) was defined as an amount of enzyme that oxidizes 1 µmol ABTS per minute.

### Determination of V_max_ and K_M_

The V_max_ and K_M_ values were determined using ABTS as a substrate in a concentration range from 1.0 to 0.0125 mmol in acetate buffer (50 mmol, pH 4.0). Michaelis-Menten non-linear regression analyses were performed with Origin8pro.

### Effects of pH under RMF exposure on laccase activity and stability

The pH effect on enzymatic activity and stability was tested using a series of ABTS (0.5 mmol) solutions at pH range from 3.0 to 6.0 (Tris-glycine, acetate and phosphate buffers, 50 mmol) instead of the standard enzyme assay. The pH stability was measured every hour for 8 hours at pH 4.0, 6.0, and 8.0. The pH stability of buffer exposed to RMF was controlled by a pH-meter (EPP-1, Elemtron, Polska).

### Statistical analysis

For statistical analysis of normality of the results, Shapiro-Wilk test was performed. Statistical analysis of the results was performed with the use of analysis of variance (ANOVA, Kruskall – Wallis test). All measurements were carried out in four sets of three repetitions (n = 12). The differences between mean values were considered significant at *p*-values < 0.05. All statistical analyses was conducted with Statistica 12 software.
